# Real-world effectiveness of an intranasal spray A8G6 antibody cocktail in the post-exposure prophylaxis of COVID-19

**DOI:** 10.1038/s41392-023-01656-5

**Published:** 2023-10-23

**Authors:** Xiaosong Li, Pai Peng, Haijun Deng, Qian Yang, Shi Chen, Benhua Li, Miao He, Aishun Jin, Zhu Yang, Ni Tang, Ailong Huang

**Affiliations:** 1 Clinical Molecular Medicine Testing Center, The First Affiliated Hospital of Chongqing Medical University, Chongqing, 400016 China; 2 Key Laboratory of Major Brain Disease and Aging Research (Ministry of Education), Institute for Brain Science and Disease, Chongqing Medical University, Chongqing, 400016 China; 3 Key Laboratory of Molecular Biology for Infectious Diseases (Ministry of Education), Department of Infectious Diseases, Chongqing Medical University, Chongqing, China; 4 Institute for Viral Hepatitis, the Second Affiliated Hospital of Chongqing Medical University, Chongqing, China; 5 Laboratory Animal Center of Chongqing Medical University, Chongqing, China; 6 Department of Immunology, College of Basic Medicine, Chongqing Medical University, Chongqing, 400010 China; 7 Chongqing Key Laboratory of Basic and Translational Research of Tumor Immunology, Chongqing Medical University, Chongqing, 400010 China; 8 Department of Gynecology and Obstetrics, the Second Affiliated Hospital of Chongqing Medical University, Chongqing, China

**Keywords:** Infectious diseases, Clinical trials

## Abstract

Previously, we identified an antibody combination A8G6 that showed promising efficacy in COVID-19 animal models and favorable safety profile in preclinical models as well as in a first-in-human trial. To evaluate the real-word efficacy of A8G6 neutralizing antibody nasal spray in post-exposure prophylaxis of COVID-19, an open-label, non-randomized, two-arm, blank-controlled, investigator-initiated trial was conducted in Chongqing, China (the register number: ChiCTR2200066416). High-risk healthy participants (18–65 years) within 72 h after close contact to COVID-19 patients were recruited and received a three-dose (1.4 mg/dose) A8G6 treatment daily or no treatment (blank control) for 7 consecutive days. SARS-CoV-2 infection occurred in 151/340 (44.4%) subjects in the blank control group and 12/173 (6.9%) subjects in the A8G6 treatment group. The prevention efficacy of the A8G6 treatment within 72 h exposure was calculated to be 84.4% (95% CI: 74.4–90.4%). Moreover, compared to the blank-control group, the time from the SARS-CoV-2 negative to the positive COVID-19 conversion was significantly longer in the AG86 treatment group (mean time: 3.4 days vs 2.6 days, *p* = 0.019). In the secondary end-point analysis, the A8G6 nasal treatment had no effects on the viral load at baseline SARS-CoV-2 RT-PCR positivity and the time of the negative COVID-19 conversion. Finally, except for 5 participants (3.1%) with general adverse effects, we did not observe any severe adverse effects related to the A8G6 treatment. In this study, the intranasal spray AG86 antibody cocktail showed potent efficacy for prevention of SARS-CoV-2 infection in close contacts of COVID-19 patients.

## Introduction

Until recently, the unprecedented COVID-19 pandemic had been declared a Public Health Emergency of International Concern by the World Health Organization. Due to the continuous evolution of SARS-CoV-2, its variants led to a high risk of COVID-19 global transmission. Although vaccination has played important roles in preventing and controlling COVID-19^1,2^, the neutralizing antibodies (NAbs) elicited by vaccines were heterogeneous among different individuals and were waning within several months^3–5^.

NAbs blocking the entry of SARS-CoV-2 into host cells have been developed for the COVID-19 prevention or therapy. Several SARS-CoV-2 targeting monoclonal antibodies (mAbs) have previously been authorized for use through an emergency use authorization (EUA)^6–10^. However, due to the failure or significant decrease of neutralization against some emerging SARS-CoV-2 variants, the usage of these antibody drugs was limited. There is an urgent need to develop broad-spectrum and effective NAbs against the circulating and other novel SARS-CoV-2 variants. Furthermore, those approved neutralizing antibodies, when administrated systemically, provided limited efficacy in the prevention of viral infection. We hypothesized that this was due to the low concentration of those neutralizing antibodies at nasal compartment when administered systemically. As a potentially more effective prophylactic approach, we proposed to use neutralizing antibodies as nasal spray to prevent viral infection at the viral entry point to human body.

A8G6 is a combination of 58G6 and 55A8 monoclonal NAbs which were identified from COVID-19 convalescent patients at early 2020^11^. Previous studies^12,13^ have shown that 58G6 recognizes both the steric site S470-495 and another region, S450-458, on the receptor binding domain (RBD) of SARS-CoV-2 spike protein (S protein). When administrated as a nasal spray, 58G6 demonstrated prophylactic efficacy against authentic SARS-CoV-2 ancestral strain and the Beta variant (B.1.351) in the transgenic mice expressing human ACE2 (hACE2) and against Delta and Omicron variants in hamster model. 55A8 exhibited potent binding affinities to the S proteins of ancestral SARS-CoV-2 strain, Delta, Omicron BA.1, BA.2, and BA.4/5 at sub-picomolar level^14^. When the two NAbs simultaneously interacted with S protein, 58G6 and 55A8 recognized different and complementary epitopes in RBD of SARS-CoV-2 and further occluded the accessibility of the S protein to ACE2. Therefore, A8G6 antibody cocktail which consisted of two potent neutralizers 58G6 and 55A8 displayed a synergetic potency and the broad neutralization against the Omicron variants^14^. In the same study, intranasal delivery of the cocktail A8G6 also demonstrated potent protection against Omicron in hamster model. We also reported a first-in-human trial of the intranasal spray A8G6 antibody cocktail in healthy volunteers. Nasal delivery of A8G6 cocktail was conducted in 108 healthy volunteers. Tolerability and pharmacokinetics (nasal and serum concentration over time) of A8G6 nasal spray were assessed. Results provided evidences for safety and the potential clinical efficacy in preventing Omicron BA.4/5 infections^15^. The real-world effectiveness of the A8G6 nasal spray needs to be further evaluated.

Here we conducted an open-label, non-randomized, two-arm, blank-controlled trial among close contacts of COVID-19 patients in several designated quarantine hotels to assessed the effectiveness and safety of A8G6 intranasal spray for the post-exposure prophylaxis of COVID-19 during the Omicron BA.5.2 wave occurred in November, 2022 in Chongqing, China.

## Results

**Fig. 1:**
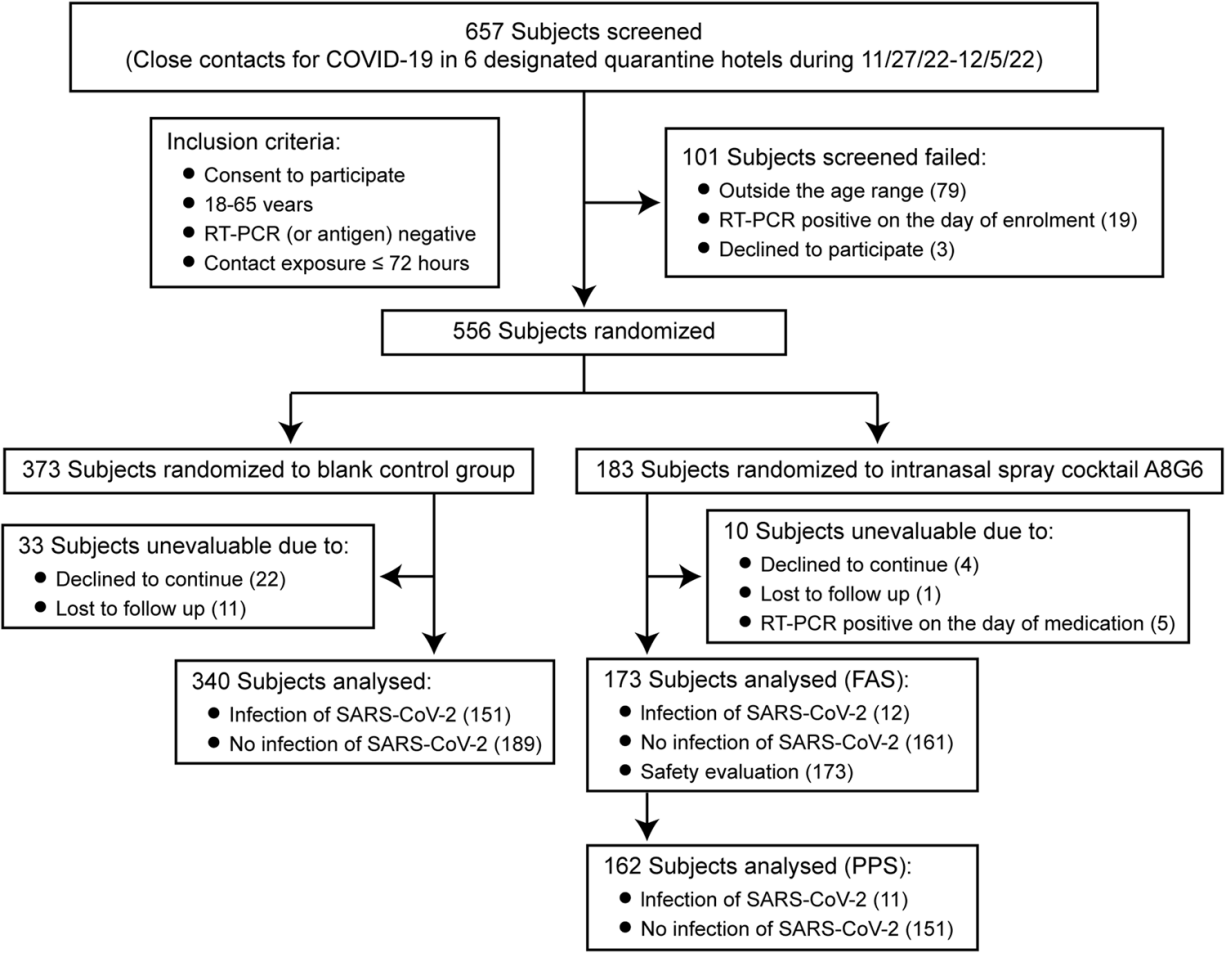
Screening and follow-up of participants. Healthy adults aged between 18 to 65 years who had a close contact with index cases within 72 h were enrolled into this study from 6 quarantine sites in Chongqing between Nov 27, 2022 and Dec 5, 2022. During this trial, the outbreak of COVID-19 was dominant by BA.5.2 (Omicron subvariant)

Since November 27, 2022, a total of 657 individuals were screened in the designated quarantine hotels. There were 101 individuals excluded according to the inclusion and exclusion criteria. The remaining 556 individuals were assigned into either A8G6 treatment group or blank-controlled group based on their preference during signing of consent form. For participants who indicated “no preference” in study group assignment, they were randomly assigned to A8G6 treatment group or the blank control group. Ten participants in the treatment group and 33 participants in the control group were excluded due to consent withdrawal or loss to follow up (Fig. 1). The full analysis set (*n* = 513) included all participants who received the A8G6 treatment (*n* = 173) or blank-control (*n* = 340) and completed the study. The per-protocol population (*n* = 162) in the treatment group was defined as individuals using A8G6 nasal spray within 72 h after exposure, while participants initially treated more than 72 h were excluded.

The final number of subjects completing the trial was 173 subjects in the A8G6 treatment group and 340 subjects in the control group. In the treatment group, 4 participants started to self-administrated A8G6 at the same day after exposure (Day 0); 73 participants used the nasal spray at the first day after exposure (Day 1); 49 participants at the second days after exposure (Day 2); 35 participants at the third day after exposure (Day 3) and 12 participants at more than 4 days after exposure (Day≥4). Among all participants in the full analysis set, median age was 36.0 (interquartile range, IQR: 26.0–48.0) years; there was a comparable sex ratio between the A8G6 group (55.5% for male and 44.5% for female) and the control group (58.2% for male and 41.8% for female); median BMI was 22.9 (IQR: 20.8–25.4); 18 (10.4%) participants in the treatment group have comorbidities, while 44 (12.9%) participants in the control group have comorbidities. 98.4% participants received different doses of COVID-19 vaccines (Table 1).

**Table 1:** Demographic and clinical characteristics of the cohort

Characteristic	A8G6 (*N* = 173)	Control (*N* = 340)	Total (*N* = 513)
*Age*			
Median age (IQR, year)	29.0 (24.0–40.0)	41.0 (30.0–50.0)	36.0 (26.0–48.0)
Mean ± sd	32.6 ± 10.4	40.1 ± 12.2	37.6 ± 12.2
*Sex*			
Male	96 (55.5%)	198 (58.2%)	294 (57.3%)
Female	77 (44.5%)	142 (41.8%)	219 (42.7%)
*Weight* (mean ± sd, kilogram)	60.0 (53.0–70.0)	63.0 (55.0–70.0)	63.0 (55.0–70.0)
*BMI* (mean ± sd)	22.2 (19.6–24.5)	23.1 (21.3–25.6)	22.9 (20.8–25.4)
*Comorbidities*			
Metabolic disease	6 (3.5%)	28 (8.2%)	34 (6.6%)
Respiratory disease	3 (1.7%)	7 (2.1%)	10 (1.9%)
Cardiovascular diseases	1 (0.6%)	1 (0.3%)	2 (0.4%)
Other	8 (4.6%)	8 (2.4%)	16 (3.1%)
Any	18 (10.4%)	44 (12.9%)	62 (12.1%)
*Vaccine types*			
Inactivated vaccine (SinoVac or Sinopharm)	156 (90.2%)	317 (93.2%)	473 (92.2%)
Recombinant vaccine (ZFLongkema)	12 (6.9%)	17 (5.0%)	29 (5.7%)
Inactivated+Recombinant vaccine	3 (1.7%)	1 (0.3%)	4 (0.8%)
Unvaccinated	2 (1.2%)	5 (1.5%)	7 (1.4%)
*Dose*			
0-dose	2 (1.2%)	5 (1.5%)	7 (1.4%)
1-dose	0 (0.0%)	5 (1.5%)	5 (1.0%)
2-dose	30 (17.3%)	42 (12.4%)	72 (14.0%)
3-dose	140 (80.9%)	286 (84.1%)	426 (83.0%)
4-dose	1 (0.6%)	1 (0.3%)	2 (0.4%)
Missing	0 (0.0%)	1 (0.3%)	1 (0.2%)
*Duration from last vaccination to exposure (day)*	398.5 (346.5–488.0)	387.0 (306.0–459.2)	389.0 (311.2–462.8)
*COVID-19 outcome*			
Positive (*n*, %)	12 (6.9%)	151 (44.4%)	163 (31.8%)
Negative (*n*, %)	161 (93.1%)	189 (55.6%)	350 (68.2%)

Shown are all participants who were recruited in our trail and received A8G6 treatment or no treatment. *BMI* denotes body mass index, Control denotes blank control without any treatment, while other participants received the A8G6 treatment; *IQR* denotes interquartile range

### Efficacy of A8G6 nasal spray in the post-exposure prevention of SARS-CoV-2 infection

**Fig. 2:**
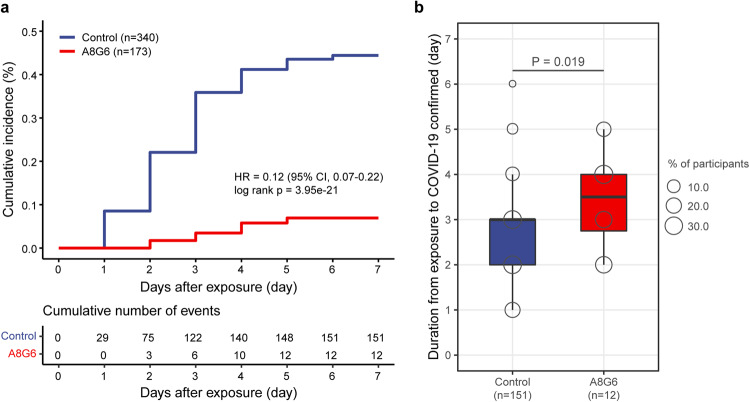
Kaplan–Meier plot of occurrence of RT-PCR-confirmed COVID-19. Shown are the primary endpoint of this trial: cumulative incidence of COVID-19 from exposure(**a**) and days from exposure to COVID-19 confirmed (**b**) in the full analysis population (*n* = 513). COVID-19 patients were defined by RT-PCR tests of oropharyngeal swab or rapid antigen tests. The COVID-19 incidence was analyzed using the Kaplan–Meier method and log-rank test. The time from exposure to confirmed SARS-CoV-2 infection was analyzed using Wilcoxon rank-sum test

After enrollment, oropharyngeal swabs of all subjects in the full analysis set were taken for RT-PCR test for SARS-CoV-2 infection every day. In total, 163/513 (31.8%) participants developed COVID-19 during the 14-day follow-up study. Among them, 12/173 (6.9%) individuals were in the A8G6 treatment group and 151/340 (44.4%) were in the blank control group (Tables 1–3, Table S1 and Fig. 2a). This difference in COVID-19 incidence rate between groups was statistically significant (Hazard ratio, HR = 0.12, 95% CI, 0.07–0.22; log-rank *p* < 0.001). The mean ( ± SD) time of the positive COVID-19 conversion was significantly longer in the A8G6 group compared to the control group (3.4 ± 1.1 days vs 2.6 ± 1.2 days, *p* = 0.019) (Fig. 2b). Similar results of data analysis were obtained in the per protocol set (data not shown).

**Table 2:** Demographic and clinical characteristics of COVID-19 positive Individuals

Characteristic	A8G6 (*N* = 12)	Control (*N* = 151)	*P* value
*Age*			0.103
Median age (IQR, year)	36.0 (31.2–45.5)	42.0 (32.0–51.0)	
Mean ± sd	36.3 ± 10.4	41.8 ± 12.0	
*Sex*			0.375
Male	8 (66.7%)	77 (51.0%)	
Female	4 (33.3%)	74 (49.0%)	
*Weight (mean* *±* *sd)*	61.5 (58.8–66.2)	63.0 (55.0–70.0)	0.934
*BMI (mean* *±* *sd)*	21.4 (20.9–23.2)	23.7 (21.4–26.0)	0.057
*Clinical phenotype*			0.694
Symptomatic	11 (91.7%)	127 (84.1%)	
Asymptomatic	1 (8.3%)	24 (15.9%)	
*Duration from exposure to COVID-19 confirmed (day)*			0.019
median days, IQR	3.5 (2.8–4.0)	3.0 (2.0–3.0)	
Mean ± sd	3.4 ± 1.1	2.6 ± 1.2	
*Duration of SARS-CoV-2 positive (day)*			0.724
median days, IQR	6.5 (5.0–7.2)	7.0 (4.0–7.0)	
Mean ± sd	6.7 ± 1.9	6.3 ± 2.5	
*Viral load (Conversion according to Ct value) of ORF1ab gene)*			1.000
High level, >10^5^ copies/ml	5 (41.7%)	69 (45.7%)	
Low level, <10^5^ copies/ml	7 (58.3%)	82 (54.3%)	
*Viral load (Conversion according to Ct value) of N gene)*			0.117
High level, >10^5^ copies/ml	5 (41.7%)	100 (66.2%)	
Low level, <10^5^ copies/ml	7 (58.3%)	51 (33.8%)	
*Symptomatic treatment*			0.039
Western medicine	11 (91.7%)	89 (58.9%)	
Traditional chinese medicine	0 (0.0%)	8 (5.3%)	
Combination of western and traditional chinese medicine	1 (8.3%)	6 (4.0%)	
Untreated	0 (0.0%)	48 (31.8%)	
*Duration of Covid-19 symptoms (d)*			0.401
median days, IQR	5.0 (1.5–5.0)	3.0 (2.0–4.5)	
Mean ± sd	4.3 ± 2.9	4.2 ± 5.0	
*Comorbidities*			0.435
Metabolic disease	1 (8.3%)	17 (11.3%)	
Respiratory disease	1 (8.3%)	2 (1.3%)	
Cardiovascular diseases	0 (0.0%)	1 (0.7%)	
Other	1 (8.3%)	5 (3.3%)	
All	3 (25.0%)	25 (16.6%)	
*Vaccine type*			0.203
Inactivated vaccine	10 (83.3%)	143 (94.7%)	
Recombinant vaccine	2 (16.7%)	5 (3.3%)	
Unvaccinated	0 (0.0%)	3 (2.0%)	
*Dose*			1.000
0-dose	0 (0.0%)	3 (2.0%)	
1-dose	0 (0.0%)	1 (0.7%)	
2-dose	2 (16.7%)	24 (15.9%)	
3-dose	10 (83.3%)	123 (81.5%)	
*Duration from last vaccination to exposure (day)*	359.5 (272.2–456.2)	384.0 (299.5–463.0)	0.622
*Signs and symptoms*^*a*^			
Fever	10 (83.3%)	83 (55.0%)	0.071
Fatigue	4 (33.3%)	22 (14.6%)	0.102
Dry cough	6 (50.0%)	54 (35.8%)	0.361
Headache	3 (25.0%)	39 (25.8%)	1.000
Dizziness	0 (0.0%)	5 (3.3%)	1.000
Ageusia	2 (16.7%)	8 (5.3%)	0.161
Pharyngalgia	1 (8.3%)	12 (7.9%)	1.000
Myalgia	2 (16.7%)	37 (24.5%)	0.733
Chill	1 (8.3%)	3 (2.0%)	0.266

Shown are COVID-19 positive participants who received A8G6 treatment or no treatment. *BMI* denotes body mass index; Control denotes blank control without any treatment, while other participants received the A8G6 treatment, *IQR* denotes interquartile range, *Ct* denotes cycle threshold

^a^Due to the limit space, more signs and symptoms were removed into an individual table as supplementary materials (supplementary Table. S2)

**Table 3:** Primary and key secondary efficacy end points

End points	A8G6 (*n* = 178)	Control (*n* = 340)
*Primary end point*		
SARS-CoV-2 confirmed by RT-qPCR		
No. of participants (%)	12 (6.9%)	151 (44.4%)
Hazard ratio (95% CI)	0.12 (0.07–0.22)	-
log rank *P* value	3.95E-21	-
Days to SARS-CoV-2 confirmed		
Total No. of days	41	392
Mean days to SARS-CoV-2 confirmed (days)	3.4	2.6
*P* value	0.019	-
*Key secondary end points*		
High viral load at SARS-CoV-2 confirmed, ORF1ab > 10^5^ copies/ml		
No. of participants (%)	5 (41.7%)	69 (45.7%)
*P* value	1.000	-
High viral load at SARS-CoV-2 confirmed, *N* > 10^5^ copies/ml		
No. of participants (%)	5 (41.7%)	100 (66.2%)
*P* value	0.117	-
SARS-CoV-2 negative conversion		
No. of participants (%)	12 (100.0%)	151 (100.0%)
Hazard ratio (95% CI)	0.98 (0.54–1.77)	-
log rank *P* value	0.946	-
Duration of SARS-CoV-2 positive (day)		
Total No. of days	80	917
Mean days of SARS-CoV-2 positive duration	6.7	6.3
*P* value	0.724	-

Shown are primary and key secondary efficacy of the intranasal spray A8G6 antibody cocktail. All participants who were recruited in our trail and received A8G6 treatment or no treatment (in the control group). *CI* denotes confidence interval

### The effect of A8G6 on the viral load of SARS-CoV-2 infection at baseline

**Fig. 3:**
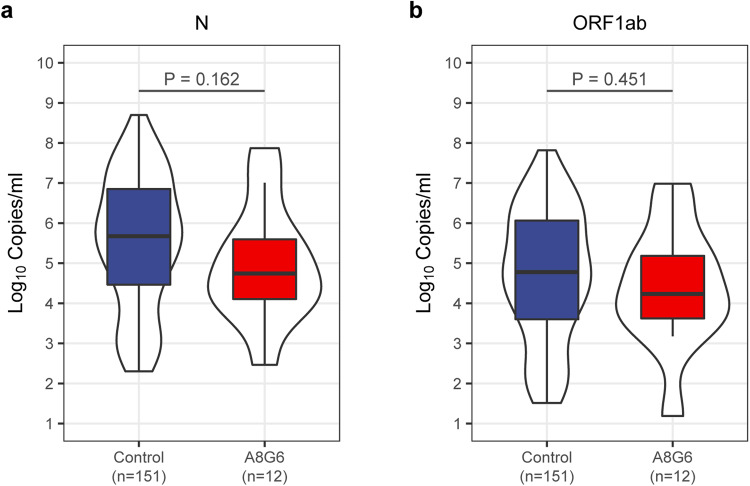
SARS-CoV-2 viral load (log10 copies per ml) at baseline when diagnosed with COVID-19. Shown are SARS-CoV-2 viral copies of COVID-19 confirmed participants in the full analysis population (*n* = 173), which were presented by converting from the Ct values of N gene (**a**) and ORF1ab gene (**b**). The viral load was analyzed using Wilcoxon rank-sum test

After enrollment, oropharyngeal swabs of all subjects were taken for RT-PCR test for SARS-CoV-2 every day. When participants were diagnosed as SARS-CoV-2 infection, the Ct values of ORF1ab and N genes were recorded and converted into copies per mL log10 values. Five subjects (41.7%) in the A8G6 treatment group had high viral load ( > 10^5^ copies/ml) of the ORF1ab gene, compared with 69 subjects (45.7%) in the control group (Table 3); Five subjects (41.7%) in the A8G6 treatment group had high viral load of the N gene ( > 10^5^ copies/ml), compared with 100 subjects (66.2%) in the control group. There were no significant differences on the percentage of participants with high viral load of these two genes (*p* = 1.000 for ORF1ab gene and 0.117 for N gene, respectively) between the two groups. That is, despite participants received the A8G6 treatment, when they became infected with SARS-CoV-2, they had a comparable level of viral load as the infected participants in the blank control group (Fig. 3a, b). The same analysis conducted in the per protocol set obtained the consistent results (Supplementary Fig. S1a and b).

### The effect of A8G6 on the time to the COVID-19 recovery

**Fig. 4:**
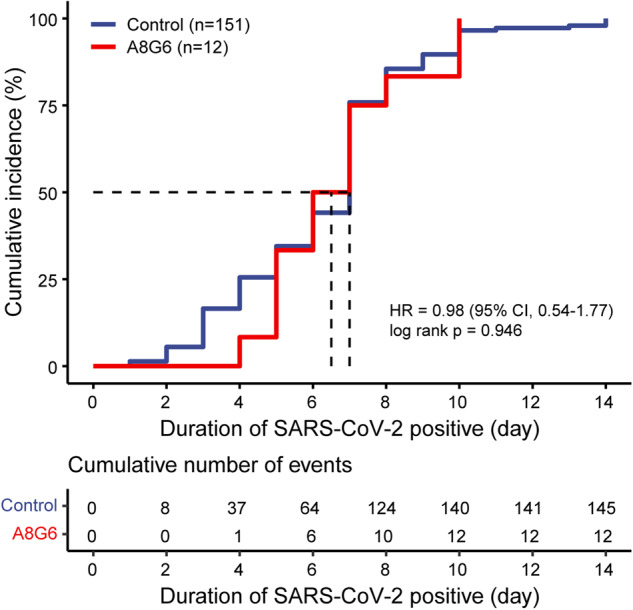
Time-to-event curve for time to viral clearance of SARS-CoV-2 in the full analysis population. Shown are cumulative incidence of COVID-19 negative conversion. Viral clearance was defined as conversion of SARS-CoV-2 RNA from positive to negative. There were 6 individuals in the control group with uncertain time of conversion of SARS-CoV-2 RNA from positive to negative. Negative conversion of SARS-CoV-2 was conducted by using Kaplan–Meier method and log-rank-test

When participants became infected with SARS-CoV-2 in both groups, RT-PCR tests or rapid antigen tests of their oropharyngeal swabs for COVID-19 and the COVID-19 related symptoms were continuously monitored and recorded. When SARS-CoV-2 RNA or SARS-CoV-2 related antigens could not be detected, it was defined as COVID-19 negative conversion. All subjects in both groups who became infected with SARS-CoV-2 during the trial period were observed the conversion to COVID-19 negative by the end of the trial. The time of SARS-CoV-2 negativity between groups showed no statistical differences (*p* = 0.946) (Fig. 4). There is a similar result in the per protocol set (Supplementary Fig. S2).

### Safety

Participants receiving A8G6 treatment (*n* = 173) were required to recorded adverse events (AEs). AEs reported by COVID-19 negative participants (*n* = 161) were not correlated with COVID-19, but might be correlated with the A8G6 treatment. AEs reported by COVID-19 positive participants (*n* = 12) might be correlated with COVID-19 or A8G6. Therefore, after the exclusion of AEs related to COVID-19, the presumptive AEs related to A8G6 treatment were analyzed. Total of 96.9% of the participants in the A8G6 treatment group had no treatment-related adverse effects. Only 3.1% subjects reported one adverse event. The specific performance included nasal swelling (*N* = 2, 1.24%), dry throat (*N* = 2, 1.24%) and ageusia (*N* = 1, 0.62%). No adverse events of special interest were reported during the trial period, and no participants withdrew from the trial because of an adverse event. There is a similar result in the per protocol set (Supplementary Table. S1).

## Discussion

The nasal spray antibody cocktail A8G6 had demonstrated broad spectrum potency blocking the SARS-CoV-2 infection in our previous preclinical data and also demonstrated favorable safety profile in a first-in-human trial (unpublished data, manuscript in preparation). In this study, we conducted an open-label, non-randomized, two-arm, blank-controlled trial among close contacts of COVID-19 patients in several designated quarantine hotels, during the COVID-19 outbreak occurred in November, 2022 in Chongqing, China. The intranasal spray antibody cocktail A8G6 was assessed to the effectiveness and safety for the post-exposure prophylaxis of COVID-19 in the real-world. Our data suggest that the application of A8G6 in the close contacts within the 72 h exposure decreased COVID-19 incidence rate by more than 30%. Moreover, the A8G6 treatment delayed the occurrence of SARS-CoV-2 infection by at least one day.

At present, most previously authorized COVID-19 antibody treatments under EUA were administrated via vein or intramuscular injection with a high dosage. Those treatment also had several adverse effects that affect quality of life, including pain at the site of injection, allergic reaction, nausea and so on^16^. As a respiratory pathogen, SARS-CoV-2 infection is primarily caused by breathing in infectious viral particles through nasal airway. An intranasal spray of neutralizing antibodies may provide a more direct protection against viral entry. Moreover, this non-invasive drug delivery is easier to use and may result in better medication compliance. In our study, the favorable safety profile of A8G6 with the few adverse effects was consistent with other nasal spray drugs^17^. Thus, A8G6 can be used in a wide range of population, especially in some special population with comorbidities and immunocompromised population. The effective treatment of A8G6 among high-risk patients could reduce medical cost, usage of medical resources and COVID-19 transmission risk. Furthermore, participants who experienced SARS-CoV-2 infection under the A8G6 treatment, showed delayed COVID-19 infection by ~ 1 day, which could provide important relief on medical resources at the epidemic peak. Currently, there are a few other antibody nasal sprays in clinical development. The neutralization efficacy of the nasal spray of a monoclonal antibody 35B5 against SARS-CoV-2 variants within 48 and 72 h after treatment was calculated as 60 and 20%, respectively^18^. The effectiveness of the SA58 nasal spray was evaluated as 77.7% (95% CI: 52.2–89.6%) and 61.83% (95% CI: 37.5–76.69%) in medical personnel and healthy workers (healthy adults working at construction sites), respectively^19,20^. In our primary endpoint analysis, the nasal spray A8G6 antibody cocktail showed decreased risk of infection of close contacts with COVID-19 patients. The prevention efficacy of the A8G6 treatment within 72 h exposure was calculated to be 84.4% (95% CI: 74.4–90.4). A8G6 showed comparable or better COVID-19 prevention in the real world than other similar antibody nasal spray.

Current data in this study showed that 6.9% of A8G6 treated participant became SARS-CoV-2 positive (vs 44.4% in the blank control group) during the study period. Our results suggested that post-infection A8G6 treatment provided limited benefits on viral load reduction and time to viral clearance. This is consistent with the potential mechanism of action of A8G6 nasal spray. Once SARS-CoV-2 virus enters into the cells and starts viral replication, A8G6 neutralizing antibody has limited efficacy to stop the viral replication. Our data also indicated that the efficiency of viral replication of these two groups were similar^21^. In another study, the similar viral load was also reported between the vaccinated individuals with breakthrough infections and unvaccinated individuals with SARS-CoV-2 infection ^22^.

There were several limitations for this study. First limitation is the lack of a placebo arm. We did not conduct this study with the double-blind procedure because there was a small window of time to initiate and complete the study so not allowing enough time for the placebo to be produced before the trial. Second limitation is the lack of participant randomization in the study design. This was primarily due to a large percentage of eligible participants, especially older people, showed their unwillingness or worries to take the A8G6 treatment at the time of enrollment. Therefore, we had to assign those participants to blank-controlled group. Under this situation, complete randomization was impossible. However, we supposed that our data and conclusion were not affected by ages of these participants. Because in our previous first-in-human trial, pharmacokinetics of A8G6 nasal spray showed that A8G6 has minimum penetration in the systemic blood circulation. Neutralizing activity of A8G6 against SARS-CoV-2 focuses on nasal mucosa, which is less affected by age. Third limitation is the lack of participants developing severe COVID-19 that need hospitalization due to small sample size. Therefore, this study did not assess the efficacy of A8G6 in preventing severe COVID-19. During the study period, there was an adjustment of the public health policy of the COVID-19 pandemic in China, that the SARS-CoV-2 infected persons no longer were reported in the future. As a result, the definition of close contacts became difficult and it became difficult to enroll more participants to increase the sample size. Fourth limitation is that the study was conducted in the designated quarantine hotels. Study participants were assumed to be single-exposure to positive COVID-19 individuals. The effects of increased infection risks of multiple exposures in the real world on the A8G6 efficacy should be considered in the further study.

In conclusion, we observed potent post-exposure prevention efficacy of intranasal spray AG86 antibody combination in close contacts of COVID-19 patients. This proof-of-concept study result suggested the potential beneficial effect of neutralizing antibody administrated as nasal spray in COVID-19 prevention. Currently A8G6 nasal spray is under clinical development to further assess its efficacy and safety.

## Materials and methods

### Study design

In this study, an open-label, non-randomized, two-arm, blank-controlled, investigator-initiated trial was designed to assess the efficacy and safety of the intranasal spray cocktail A8G6 in preventing SARS-CoV-2 infection among close contacts with COVID-19 patients. The clinical trial was conducted at 6 designated quarantine hotels in Yuzhong District, Chongqing, China from November 27, 2022 and was completed on December 12, 2022.

Recruited participants in the treatment group self-administrated a three doses of 0.7 mg (140 μl) A8G6 nasal spray per day for 7 treatment days. The drug was supplied by Chongqing Mingdao Haoyue Biotechnology Co., LTD (Chongqing, China), stored at 2–8 °C. In the blank control group, participants did not receive any treatment. After enrollment, SARS-CoV-2 infection was confirmed by a reverse transcription polymerase chain reaction (RT-PCR) test of oropharyngeal swab. During this trial, with the adaption of the anti-COVID-19 policy, not only RT-PCR, but also rapid antigen tests were used to confirm the SARS-CoV-2 infection status.

The trial was carried out in accordance with all applicable national and local regulatory requirements. Data and Safety Monitoring Board of The Second Affiliated Hospital of Chongqing Medical University oversaw trial conduct and documentation. The protocol has been approved by the Chinese clinical test registration center (the world health organization international clinical trials registered organization registered platform (ICTRP), the registration number: ChiCTR2200066416) and the Ethics Committees of The Second Affiliated Hospital of Chongqing Medical University (the approval number: 2022127-1).

### Participants

During November COVID-19 wave in Chongqing, China, when patients had been diagnosed as COVID-19 with the positive RT-PCR test for SARS-CoV-2 (index cases), their close contacts were immediately transferred to the designated quarantine sites. At 6 quarantine sites in Chongqing, healthy adults aged between 18 to 65 years who had a close contact with index cases within 72 hours were enrolled into this study. The maximum time interval between exposure to treatment was ≤72 h. All vaccination status is eligible for inclusion. Exclusion criteria included positive RT-PCR at baseline, nasal discomfort, the use of other COVID-19 antibody drugs and drug-drug interference with participants’ regular medication (additional details about eligibility criteria were described in the appendix).

All study participants were capable of self-administrating the intranasal spray, recording and recalling clinical signs. All participants were provided and voluntarily signed written informed consent before the study.

### Procedures

At six quarantine sites in the Yuzhong District, Chongqing, site investigation was carried out to screen eligible participants. Eligible participants were given the choice to join the A8G6 treatment group or blank control group. For eligible participants that showed “no preference” in either group, they were randomly assigned to A8G6 treatment group or blank control group. Oropharyngeal swabs were taken for quantitative and qualitative RT-PCR assessments at baseline prior to treatment and though the treatment period and a follow-up period. Subjects with positive RT-PCR results before treatment were excluded. The SARS-CoV-2 viral load was present by viral genome copies per mL log10 values with the conversion of the open reading frame of 1ab (ORF1ab) and nucleocapsid (N-gene) cycle threshold (Ct) values (RT-PCR was conducted by Yuzhong District Center for Disease Control and Prevention, in Chongqing, China. Conversion of Ct values to viral genome copies was calculated according to the manufacturer’s instructions of 2019-nCoV viral RNA kit produced by BioPerfectus Technologies, catalog number: JC10223-1N).

Subjects’ demographic data, health and COVID-19 vaccination status were recorded at the baseline visit (Day 0). The use of nasal spray, rapid antigen tests or RT-PCR test for COVID-19 were recorded every day during the study participation. When participants in both groups were diagnosed with SARS-CoV-2 infection, the related symptoms and symptomatic treatment for COVID-19 were reported until the trial completed. In the treatment group, all participants were requested to self-report and record the adverse events. Due to the relaxation of COVID-19 control and policy starting from December 4, 2022, some participants returned to home for further isolation. The follow-up visits were adjusted to retrospective telephonic visit according to a questionnaire form from that day.

### Outcomes

The primary endpoint analysis included all participants in both the treatment and control groups. The primary endpoint was to assess the efficacy of the intranasal spray A8G6 for post-exposure prophylaxis of COVID-19. In this study, we compared the COVID-19 incidence of the close contacts between the A8G6 treatment individuals and the blank-controlled individuals. We also compared the time from enrollment to SARS-CoV-2 infection between the two groups. The secondary efficacy analysis included the quantitative data of SARS-CoV-2 RNA (log10 copies per mL) at baseline of the positive COVID-19 and the time to conversion of SARS-CoV-2 RNA from positive to negative (viral clearance).

Safety endpoints was adverse event types and the incidence rate of adverse events among all participants of the A8G6 treatment group during the study. An adverse effect was defined as any abnormal signs or symptoms and harmful results caused by the study drug.

### Statistical analysis

The sample size in this clinical trial was determined on the basis of statistical power calculations. We proposed greater than 90% power to detect a 20% relative difference between the A8G6 treated and control group at a two-sided alpha level of 0.05 (ie., a 20% prevention efficacy of A8G6). The formula is as follows:

$$n={\frac{2pq({Z}_{1-\frac{\alpha }{2}}+{Z}_{1-\beta })}{{\delta }^{2}}}^{2}$$

which *p* is the proportion of participants develop COVID-19 in A8G6 treated group, q is in the control group, δ is the difference between two group, α is two-sided alpha level, and 1-β is statistical power. In this clinical trial, we assume that q is 0.1, 20% relative reduction of A8G6 treated group is 0.08. Assuming a dropout rate of 20%, at total of 5160 participants will be recruited.

The primary efficacy endpoints including COVID-19 incidence and time to confirmed SARS-CoV-2 infection. The COVID-19 incidence was analyzed using the Kaplan–Meier method and log-rank test, and the time to confirmed SARS-CoV-2 infection was analyzed using Wilcoxon rank-sum test. The secondary efficacy endpoints including viral load when confirmed SARS-CoV-2 infection and the time to negative conversion of SARS-CoV-2 determined by RT-PCR. The viral load when confirmed SARS-CoV-2 infection was analyzed using Wilcoxon rank-sum test, negative conversion of SARS-CoV-2 and remission time were conducted using Kaplan–Meier method and log-rank-test. Safety was assessed in participants in the full analysis set who received A8G6 nasal spray treatment during the 8-day quarantine period.

Database from the Service Platform for COVID-19 Prevention and Control created by Yuzhong District Center for Disease Control and Prevention were authorized for us to use and analyze. Data including demographic and clinical characteristics of the cohorts, endpoints in this clinical trial were collected from an applet of WeChat (a social media platform in China), called “Yuzhong Information Exchange”. All data were summarized with descriptive statistics (number of subjects (%), median (IQR), mean ± sd). The credible interval for nasal spray was calculated with the use of a beta-binomial model with prior beta (1, 1) adjusted for the treatment duration time. Continuous variables were compared with the Mann–Whitney U-test, and Categorical variables were conducted using χ2 test or Fisher’s exact test. A *P* value of < 0.05 was considered statistically significant. Statistical analyses were performed using R software, version 3.6.0.

### Supplementary information

Supplementary materials

## Data Availability

De-identified individual participant-level data will be available upon written request to the corresponding author following publication.
