# Supplementary material for: Real-world effectiveness of an intranasal spray A8G6 antibody cocktail in the post-exposure prophylaxis of COVID-19
Source: Signal Transduct Target Ther. 2023 Oct 23;8:403. doi: 10.1038/s41392-023-01656-5 (PMC10590774; doi:10.1038/s41392-023-01656-5)
Supplement: Supplementary file 1 — Supplementary materials [file 41392_2023_1656_MOESM1_ESM.docx]

Supplementary Materials for

Real-world effectiveness of an intranasal spray A8G6 antibody cocktail in the post-exposure prophylaxis of COVID-19

Xiaosong Li*, Pai Peng2*, Haijun Deng*, Qian Yang, Shi Chen, Benhua Li, Miao He, Aishun Jin, Zhu Yang, Ni Tang#, Ailong Huang#

Correspondence to: [ahuang@cqmu.edu.cn](mailto:ahuang@cqmu.edu.cn) and [nitang@cqmu.edu.cn](mailto:nitang@cqmu.edu.cn)

**This PDF file includes:**

- Supplementary Table S1 to S2
- Supplementary Figure S1 to S2
- Supplementary Methods (The study protocol)

**Table of Content**

[**Supplementary Tables & Figures** 3](#_Toc139554262)

[Table S1. Participants with adverse events after the AG86 treatment in the per protocol population (n=150). 3](#_Toc139554263)

[Table S2. Clinical signs and symptoms of COVID-19 positive individuals 4](#_Toc139554264)

[Figure S1. SARS-CoV-2 viral load of COVID-19 positive participants (log10 copies per ml) at baseline in the per protocol population. 5](#_Toc139554265)

[Figure S2. Time-to-event curve for time to viral clearance of SARS-CoV-2 in the per protocol population. 6](#_Toc139554266)

[**Supplementary methods 7**](#_Toc139554267)

[(The study protocol) 7](#_Toc139554268)

[Clinical research unit： 7](#_Toc139554270)

[Protocol Signing Page 8](#_Toc139554271)

[(Clinical research unit) 8](#_Toc139554272)

[Protocol Signing Page 9](#_Toc139554274)

[(Statistical analysis unit) 9](#_Toc139554275)

[Summary of clinical intervention protocol 10](#_Toc139554277)

[1. Research background information 15](#_Toc139554278)

[2. Research content 17](#_Toc139554281)

[3. Research purpose 17](#_Toc139554282)

[4. Research protocol 18](#_Toc139554285)

[5. Trial drug management 26](#_Toc139554312)

[6. Preservation and confidentiality of test documents 27](#_Toc139554317)

[7. Quality control and guarantee of research 28](#_Toc139554320)

[8. Data security monitoring 29](#_Toc139554325)

[9. Research progress 29](#_Toc139554326)

[10. Participants 30](#_Toc139554327)

# Supplementary Tables & Figures

**Table S1. Participants with adverse events after the AG86 treatment in the per protocol population (n=150).**

| **Adverse events** | **n (%)** |
| --- | --- |
| Nasal swelling | 2 (1.33%) |
| Dry throat | 2 (1.33%) |
| Ageusia | 1 (0.67%) |

Shown are self-reported adverse events by participants in A8G6 treatment group. AEs related to COVID-19 were excluded, the presumptive AEs related to A8G6 treatment were analyzed.

## Table S2. Clinical signs and symptoms of COVID-19 positive individuals

| **Characteristics** | **A8G6 (N=12)** | **Control (N=151)** | **P value** |
| --- | --- | --- | --- |
| **Signs and symptoms** |  |  |  |
| Fever | 10 (83.3%) | 83 (55.0%) | 0.071 |
| Fatigue | 4 (33.3%) | 22 (14.6%) | 0.102 |
| Dry cough | 6 (50.0%) | 54 (35.8%) | 0.361 |
| Headache | 3 (25.0%) | 39 (25.8%) | 1.000 |
| Dizziness | 0 (0.0%) | 5 (3.3%) | 1.000 |
| Ageusia | 2 (16.7%) | 8 (5.3%) | 0.161 |
| Pharyngalgia | 1 (8.3%) | 12 (7.9%) | 1.000 |
| Myalgia | 2 (16.7%) | 37 (24.5%) | 0.733 |
| Chill | 1 (8.3%) | 3 (2.0%) | 0.266 |
| Rhinorrhea | 0 (0.0%) | 2 (1.3%) | 1.000 |
| Nasal congestion | 0 (0.0%) | 2 (1.3%) | 1.000 |
| Diarrhea | 0 (0.0%) | 4 (2.6%) | 1.000 |
| Anorexia | 1 (8.3%) | 6 (4.0%) | 0.421 |
| Vomiting | 1 (8.3%) | 1 (0.7%) | 0.142 |
| Arthralgia | 2 (16.7%) | 4 (2.6%) | 0.063 |
| Nausea | 0 (0.0%) | 2 (1.3%) | 1.000 |
| Abdominal pain | 0 (0.0%) | 1 (0.7%) | 1.000 |
| Dry throat | 0 (0.0%) | 2 (1.3%) | 1.000 |
| Insomnia | 0 (0.0%) | 2 (1.3%) | 1.000 |
| Somnolence | 0 (0.0%) | 2 (1.3%) | 1.000 |
| Asthma | 0 (0.0%) | 1 (0.7%) | 1.000 |
| Expectoration | 1 (8.3%) | 0 (0.0%) | 0.074 |
| Eye swelling | 1 (8.3%) | 0 (0.0%) | 0.074 |
| Any | 11 (91.7%) | 127 (84.1%) | 0.694 |

Shown are clinical signs and symptoms self-reported by participants who were infected with SARS-CoV-2 after enrollment. Control denotes blank control without any treatment, while other participants received the A8G6 treatment.


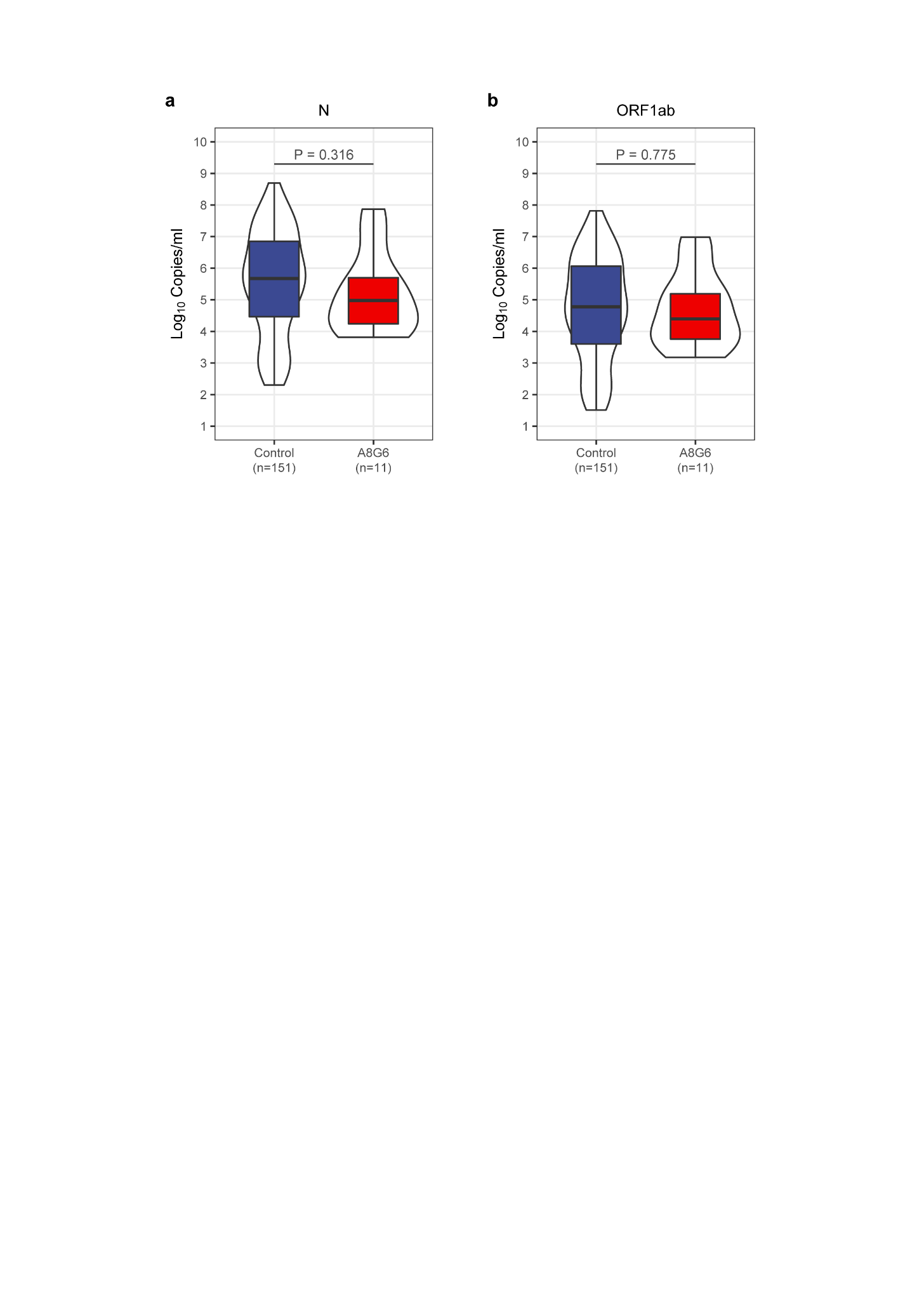


## Figure S1. SARS-CoV-2 viral load of COVID-19 positive participants (log10 copies per ml) at baseline in the per protocol population.

Shown are SARS-CoV-2 viral copies of COVID-19 confirmed participants in the per protocol population (n=162), which were presented by converting from the Ct values of N gene (a) and ORF1ab gene (b). The viral load was analyzed using Wilcoxon rank-sum test.

##
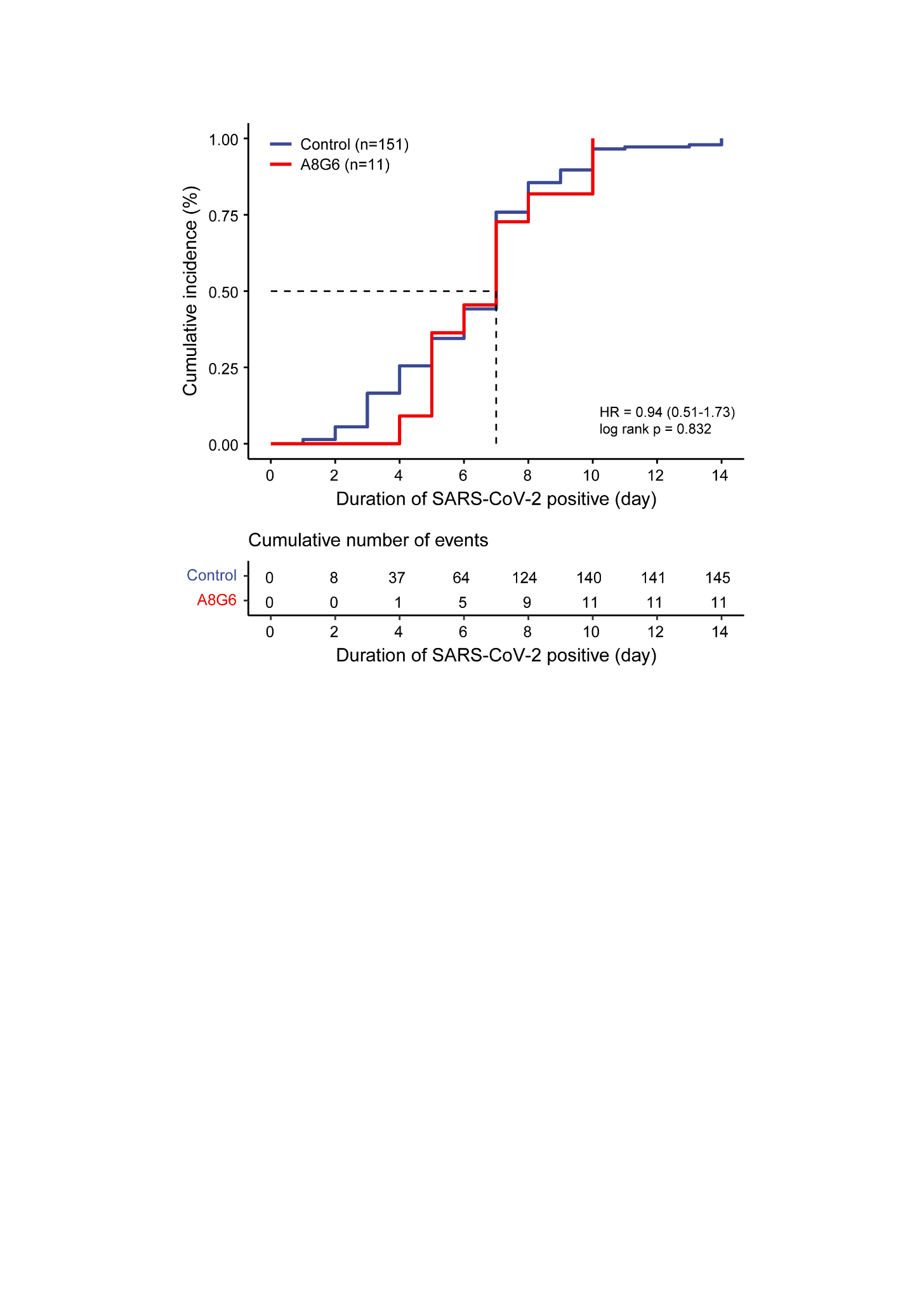


## Figure S2. Time-to-event curve for time to viral clearance of SARS-CoV-2 in the per protocol population.

Shown are cumulative incidence of COVID-19 negative conversion in the per protocol population. Viral clearance was defined as conversion of SARS-CoV-2 RNA from positive to negative. There were 6 individuals in the control group with uncertain time of conversion of SARS-CoV-2 RNA from positive to negative. Negative conversion of SARS-CoV-2 was conducted by using Kaplan-Meier method and log-rank-test.

# Supplementary methods

# (The study protocol)

**Community trial of A8G6 SARS-CoV-2 Neutralizing Antibody Combination Nasal Spray for the prevention of COVID-19 infection**

**Protocol No：AY-62-8003**

**Version No：V2.0**

**Version date：2022-11-23**

## Clinical research unit：

**The Second Affiliated Hospital of Chongqing Medical University**

Confidentiality Statement

All information contained in this protocol is owned by the Second Affiliated Hospital of Chongqing Medical University and is therefore only available for review by relevant medical institutions such as researchers, co-investigators, ethics committees and supervisory authorities. Without the written approval of the Second Affiliated Hospital of Chongqing Medical University, it is strictly prohibited to disclose any information to third parties not involved in the study, except to make necessary explanations when signing informed consent forms with potential participants in the study.

## Protocol Signing Page

## (Clinical research unit)

We have read and understood the protocol entitled "Community trial of A8G6 SARS-CoV-2 Neutralizing Antibody Combination Nasal Spray for the prevention of COVID-19 infection" (Version No: V2.0 , Version date: 2022-11-23) and agree to follow all regulations set out in the protocol.

## I agree that:

- Carry out this experiment in strict accordance with the protocol, current NMPA GCP and ICH GCP, as well as applicable regulations and guidelines, and conscientiously perform the duties of the researcher.
- I will be responsible for making clinically relevant medical decisions to ensure that patients receive appropriate treatment in the event of adverse events during the study: I am aware of the requirements for proper reporting of serious adverse events, and I will record and report these events as required. I guarantee that the data will be loaded into the case report form accurately, completely, promptly and legally.
- Keep all data related to this study in accordance with the confidentiality requirements.

Principal investigator： Clinical research unit：

Signature： Date：

## Protocol Signing Page

## (Statistical analysis unit)

We have read and understood the protocol entitled "Community trial of A8G6 SARS-CoV-2 Neutralizing Antibody Combination Nasal Spray for the prevention of COVID-19 infection" (Version No: V2.0 , Version date: 2022-11-23) and agree to follow all regulations set out in the protocol.

## I agree that：

- Complete the statistical analysis of the clinical study data in strict accordance with the protocol, current NMPA GCP and ICH GCP, and applicable regulations and guidelines, and conscientiously perform my duties to ensure that the statistical analysis of the clinical trial data meets the statistical requirements.
- Keep all data and information related to this study in accordance with the confidentiality requirements.

Project leader of statistical analysis unit： Statistical analysis unit：

Signature： Date：

## Summary of clinical intervention protocol

| **Study name** | Community trial of A8G6 SARS-CoV-2 Neutralizing Antibody Combination Nasal  Spray for the prevention of COVID-19 infection |
| --- | --- |
| **Study drug name** | A8G6 SARS-CoV-2 Neutralizing Antibody Combination Nasal Spray |
| **Program number** | **AY-62-8003** |
| **Clinical research unit** | The Second Affiliated Hospital of Chongqing Medical University |
| **Main researcher** |  |
| **Purpose of the experiment** | **Main Purpose:**  To assess the preventive effectiveness of A8G6 nasal spray for close contacts.  **Secondary purpose:**  ① To evaluate the effectiveness of A8G6 nasal spray on the Median time of nucleic acid positive conversion for close contacts;  ② To evaluate the intervention safety of A8G6 nasal spray in close contacts: the incidence and median time of adverse events;  ③To assess the effectiveness of A8G6 nasal spray for symptom control for close contacts;  ④ To evaluate close contacts medical compliance and time cost of medication . |
| **Overall design** | Community-controlled trial |
| **Number of close contacts** | A total of 5160 close contacts are planned |
| **Experimental drugs** | **Test drug:** A8G6 SARS-CoV-2 Neutralizing Antibody Combination Nasal Spray Specification: 5 mg/mL, 70uL per spray  Provided by Chongqing Mingdao Haoyue Biotechnology Co., Ltd |
| **Administered dose** | 1 spray in each nostril as 1 dose, 140uL/dose |
| **Method of administration** | 3 doses per day, each dose with an interval of about 6-8 hours, continuous use for no  more than 14 days. |
| **Research procedures** | After the close contact signs the informed consent form, registers personal information, exposure history, and regularly administer medication during centralized isolation and home isolation according to the method and dose of administration, and records health monitoring information such as medication compliance, symptoms,  adverse events, body temperature, and nucleic acid test results. |

| **Inclusion criteria** | 1 、 Close contacts in Chongqing isolation points and control areas: close contacts aged 18-65 (including critical values);  2、New entry into isolation points;  3、The last exposure time is within 72 hours;  4 、 Close contacts fully understand the purpose, quality, methods and possible adverse reflects of the trial, voluntarily participation in the trial, and sign the informed consent form before the start of the trial;  5、Women of childbearing age who used effective contraception within 2 weeks prior to enrollment had negative pregnancy test results prior to enrollment. All close contacts voluntarily agreed to use effective contraception from the time of signing the informed consent form until the end of the study (effective contraceptive measures include: oral contraceptives (excluding emergency contraceptives), injectable or implantable  contraception, extended-release topical contraceptives, hormone patches, IUDs, sterilization, abstinence, condoms(men), diaphragms, cervical caps, etc.);  6 、 The close contact agrees to only take SARS-CoV-2 sampling from oropharyngeal  swabs and will not perform nasal swab sampling except for this study;  7 、 Those who have a negative nucleic acid test for the SARS-CoV-2during the screening period;  8 、 Close contacts are able to communicate well with the investigator and understand and comply with the requirements of this study.  **(All of the above requirements must be met to be included in the trial)** |
| --- | --- |
| **Exclusion criteria** | 1、People with cognitive impairments, or inability to understand Chinese; Unable to use WeChat answerers, or other reasons to complete the study according to the protocol, or the investigator determines that it is not suitable for participants;  2、Patients who are unable to understand or follow the study procedure and sign written  informed consent;  3 、 Those who are allergic to any ingredients in this product and auxiliary materials; or those with allergies (such as those who are allergic to two or more drugs or foods);  4、history of previous SARS, SARS-CoV-2, MERS, or other human coronavirus infection or illness;  5、Those who have not previously tolerated intranasal administration;  6、Those who have used any nasal spray, snorting or other nasopharyngeal products |

|  | within 2 weeks prior to screening, or who cannot stop using such products during the test;  7、Those who have undergone any nasopharyngeal surgery within 1 year, have a  previous acute exacerbation of chronic rhinitis or have anatomical abnormalities in the nose that affect drug absorption;  8、lactating or pregnant women;  9 、 Those who are participating in other COVID-19-related clinical trials, and who are participating or plan to participate in other clinical trials during the study;  10 、 The investigators believe that the presence of any disease or condition in the close contact may put the close contact at unacceptable risk; Close contacts are unable to meet the requirements of the program; Situations that interfere with the assessment of drug response.  **(Trials are not included in any of the above)** |
| --- | --- |
| **Withdrawal criteria** | 1、Progression of close contacts to asymptomatic or confirmed cases;  2 、 The state of health does not allow continued participation in data collection, and cannot effectively ensure the safety of close contacts;  3 、 Failure to promptly handle and report suspicious and unexpected serious adverse reactions;  4、Clinical trials give drugs quality problems; 5、Fraud in the course of drug clinical trials; 6、loss to follow-up;  7、Other violations of the good clinical trial practice of drugs or the investigator believes  that the research cannot be continued. |
| **Rejection criteria** | 1 、 The selection of individual close contacts seriously violates the inclusion/exclusion criteria;  2 、 During the trial, close contacts did not comply with the test plan and had poor compliance, such as not using the test medication or unable to collect biological samples according to the requirements of the test protocol, which affected the evaluation of the  results. |
| **Termination criteria** | 1、The sponsor proposes to terminate the clinical trial;  2、The NMPA or the health administrative department requires the termination of the clinical trial. |

| **Evaluation indicators** | **Effectiveness evaluation indicators:**  Positive conversion rate, Median time of nucleic acid positive conversion  **Safety evaluation indicators:**  Incidence and median time of adverse events  **Feasibility evaluation indicators:**  Medication adherence: answer completion rate and medication time |
| --- | --- |
| **Statistical methods** | 1. **Effectiveness endpoint analysis:**   ① Nucleic acid conversion rate: Chi-square test was used to compare the nucleic acid conversion rate of the two groups at the end of isolation.  ② Median time to nucleic acid conversion: For the time of nucleic acid conversion, the survival curve will be plotted by the Kaplan-Meier method, and the 95% confidence interval of the median time will be calculated by the Brookmeyer Crowley method. The log-rank method was used to compare the differences between groups and based on Cox Scale models estimated HR for each endpoint and explored the impact of factors other than treatment groups on the differences between endpoints.  ③ Description of symptom burden: mean, standard deviation, median, maximum, minimum, P25, P75 to describe the symptoms of the survey population, frequency and percentage (N, %) to describe the proportion of severe symptoms and quality of life of patients;  ④ Comparison between groups of symptom trajectories: Mixed-effects model and generalized estimation equation were used to compare the incidence of symptoms and moderate to severe symptoms between groups.  ⑤ Symptoms return to normal levels: K-M (Kaplain-Meier) method is calculated, log-rank test compares differences between comparison groups, and recovery curves are plotted.   1. **Safety endpoint analysis:**   ① Incidence of adverse events: TheA8G6 group was compared with the control group. The Chi-square test was used to compare the incidence of adverse reactions in the population at the end of isolation between the two groups.  ② Median time of adverse events: The survival curve will be plotted using the  Kaplan-Meier method and the 95% confidence interval of the median time will be |

|  | calculated using the Brookmeyer Crowley method. The log-rank method was used to compare the differences between groups.  **3. Feasibility evaluation indicators:**  Medication adherence and time cost of medication: medication adherence and time cost in the intervention group were analyzed using mean ± standard deviation or completeness of answers, and deletion rate. |
| --- | --- |

## Research background information

- 1. **Research background**

The SARS-CoV-2 neutralizing antibody can directly bind to the envelope of the novel coronavirus to rapidly block viral infection, which has been fully validated as a safe and effective treatment. However, up to now, there are no approved antibodies to prevent infection of SARS-CoV-2 at home and abroad, and there is a lack of broad-spectrum and efficient monoclonal neutralizing antibodies to mutant strains. In particular, the Omicron variant reduced the neutralizing activity of current therapeutic monoclonal antibodies. Currently, most of the therapeutic neutralizing antibodies approved worldwide are used in combination with two antibodies. There is no reference method for neutralizing antibody administration for prophylaxis in a wide range of people, and the mainstream intravenous infusion as a prophylaxis method will lead to low compliance of the drug population.

Two main components of A8G6 SARS-CoV-2 neutralizing antibody: Both MY-586 and MY-558 were screened from peripheral blood lymphocytes of convalescent patients, from which 209 strains of SARS-CoV-2 specific antibodies were isolated. The screened MY-586 and MY-558 were antibodies with strong and effective neutralization against wild and circulating strains of British strain, Indian strain, South African strain, Indian Delta strain and Omicron strain. The Cryo-EM structure of the antibody/Omicron Spike protein complex shows that the two antibodies bind at non-overlapping sites, locking the Spike protein in the "up" conformation and preventing its binding to the hACE2 receptor. Consistent with the structural information, MY-586 and MY-558 showed a strong synergistic effect in the vitro model of pseudoviral cell infection. According to the broad neutralization efficacy of Omicron BA.4/5 and Delta strains, the optimal ratio of the two antibodies (MY-586: MY-558) was 1:4. The non-overlapping binding mode of the two antibodies also enabled the combination preparation A8G6 to have broad-spectrum neutralizing activity against various SARS-CoV-2 mutant strains.

Preclinical evaluation of the efficacy and safety of A8G6 and production of CMC support the introduction of A8G6 nasal spray into human studies. A8G6, when administered intranasally at a low dose (1mg/kg),

completely blocked infection of Omicron BA.1, a hamster virus challenge model. Preclinical toxicity studies of A8G6 have been conducted in mice (non-GLP), cynomolgus monkeys (non-GLP), and rhesus monkeys (GLP) for intranasal and intravenous administration. The results showed that the A8G6 cocktail was well tolerated and no serious adverse eventss were observed. The NOAEL of A8G6 nasal spray in rhesus monkeys is 4.5mg/kg or 16.8mg/ day (preliminary GLP toxicity study results, pending finalization). In preclinical PK studies in monkeys, the nasal half-life of A8G6 in monkeys was 12-14 hours when administered as a single daily (0.7mg) or three daily (0.7mg each) doses at 4-hour intervals. Nasal Cmax was reached 15 minutes after nasal spray and was up to ~50ug/mL after a single administration and ~ 90ug/mL after a third administration, indicating that the concentration of antibodies in the nasal cavity after repeated administration had an accumulation effect. However, the nasal A8G6 concentration decreased rapidly 24 hours after administration, suggesting that daily repeated administration was needed to maintain nasal concentration. If IC90 at Omicron BA.4/5 is used as the effective concentration, monkey PK studies have shown that a single dose can maintain nasal concentrations above IC90 for 24 hours. Systemic exposure to A8G6 was extremely low in preclinical toxicokinetics studies in monkeys.

Based on preclinical safety and PK results in monkeys, we conducted an investigator-initiated trial (IIT) in 108 healthy volunteers to study safety, tolerance, and PK of A8G6 in humans. A8G6 was administered intranasally as a single dose or 2-4 doses daily for 1, 3, 7, and 14 days (0.7mg per dose). This dose was well tolerated and no serious side effects were observed. Similar to preclinical PK studies in monkeys, systemic exposure to A8G6 was extremely low. One or two daily doses of A8G6 can maintain the IC90 concentration covering Omicron BA.4/5 for up to 8 hours, but not 16-20 hours after administration. Four daily doses of A8G6 provide 24-hour full day coverage much higher than the IC90 concentration of Omicron BA.4/5. Intranasal pharmacokinetic results showed that 3 doses per day provided 24 hours full coverage of IC90 concentrations higher than Omicron BA.4/5, and this design will be studied in phase 1 healthy close contacts.

In summary, the preliminary data showed that A8G6 antibody had excellent efficacy, safety and druggability. In particular, the A8G6 neutralizing antibody is administered by nasal spray. Although there are no approved nasal spray neutralizing antibody drugs on the market at home and abroad, we have successfully solved the drugging of A8G6 antibody by nasal spray and the development of nasal spray device. The nasal spray type of A8G6 neutralizing antibody is convenient to carry, easy to administer, and has strong accessibility and high compliance among the population. It can be used as a new and widely used, a safe and effective preventive measure in addition to vaccines. Therefore, rapid clinical research and development of A8G6 antibody will provide a more effective guarantee for social safety and effective prevention of COVID-19. Phase II and Phase III clinical trials have preliminarily validated and confirmed the safety and efficacy of A8G6 in a randomized controlled trial population. But it has not yet been tested in a large community trial. In this study, the preventive effect of A8G6 on positive rotation among close contacts was evaluated. Community trial design was adopted, and the close contact isolation points or communities were divided into groups to compare the positive rotation rate, positive rotation time, adverse reactions, symptoms and other indicators of the two groups of close contacts during 5+3 isolation management. In order to verify the A8G6 emergency use after exposure, the protective effect of close contact.

**1.2 Drug introduction**

【 Drug name 】

Common name: A8G6 SARS-CoV-2 Neutralizing Antibody Combination nasal spray English name:A8G6 SARS-CoV-2 Neutralizing Antibody Combination Nasal Spray Pinyin: A8G6 Xin Guan Zhong He Kang Ti Lian

【 Component 】

The main ingredients of this product are: MY-586, MY-558

【 Character 】

This product is a clear liquid from colorless to light yellow

【 Specification 】

5 mg/mL

【 Usage and dosage 】

The patient received one dose of spray in each nostril.

【 Taboo 】

Patients allergic to A8G6 antibody can not use this product again.

Allergy to A8G6 antibody active substance or any component is prohibited.

【 Note 】

Keep medications away from your eyes and ears.

【 Storage conditions 】

Store at 2~8℃.

【 Packaging 】

Celine bottle (medium borosilicate), nasal liquid medicinal spray pump.

【 Providing enterprise 】

Chongqing MingDaoHaoYue Biotechnology Co., Ltd

## Research content

To evaluate the effect of A8G6 SARS-CoV-2 Neutralizing Antibody Combination nasal spray(hereinafter referred to as A8G6 nasal spray) on the prevention of positive reversion in close contact personnel, a community trial design will be adopted, and the positive reversion rate, positive reversion time, adverse events, symptoms and other indicators of the two groups of close contact patients during 5+3 isolation management will be compared. In order to verify the A8G6 emergency use after exposure, the protective effect of close contact.

## Research purpose

## Main purpose:

To evaluate the preventive effectiveness of A8G6 nasal spray for close contact personnel.

## Secondary purpose:

To evaluate the effectiveness of A8G6 nasal spray on the Median time of nucleic acid positive conversion for close contacts;

To evaluate the intervention safety of A8G6 nasal spray in close contacts: the incidence and median time of adverse events;

To assess the effectiveness of A8G6 nasal spray for symptom control for close contacts; To evaluate close contacts medical compliance and time cost of medication .

## Research protocol

## Research object

1. **Inclusion criteria: all of the following requirements must be met to be included in the test**
   1. Close contacts in Chongqing isolation points and control areas: close contacts aged 18-65 (including critical values);
   2. New entry into isolation points;
   3. The last exposure time is within 72 hours;
   4. Close contacts fully understand the purpose, quality, methods and possible adverse reflects of the trial, voluntarily participation in the trial, and sign the informed consent form before the start of the trial;
   5. Women of childbearing age who used effective contraception within 2 weeks prior to enrollment had negative pregnancy test results prior to enrollment. All close contacts voluntarily agreed to use effective contraception from the time of signing the informed consent form until the end of the study (effective contraceptive measures include: oral contraceptives (excluding emergency contraceptives), injectable or implantable contraception, extended-release topical contraceptives, hormone patches, IUDs, sterilization, abstinence, condoms(men), diaphragms, cervical caps, etc.);
   6. The close contact agrees to only take SARS-CoV-2 sampling from oropharyngeal swabs and will not perform nasal swab sampling except for this study;
   7. Those who have a negative nucleic acid test for the SARS-CoV-2during the screening period; 8)Close contacts are able to communicate well with the investigator and understand and comply with

the requirements of this study.

## Exclusion criteria: if any of the above criteria are met, they shall not be included in the test

- 1. People with cognitive impairments, or inability to understand Chinese; Unable to use WeChat answerers, or other reasons to complete the study according to the protocol, or the investigator determines that it is not suitable for participants;
  2. Patients who are unable to understand or follow the study procedure and sign written informed

consent;

- 1. Those who are allergic to any ingredients in this product and auxiliary materials; or those with allergies (such as those who are allergic to two or more drugs or foods);
  2. history of previous SARS, SARS-CoV-2, MERS, or other human coronavirus infection or illness; 5)Those who have not previously tolerated intranasal administration;

1. Those who have used any nasal spray, snorting or other nasopharyngeal products within 2 weeks prior to screening, or who cannot stop using such products during the test;
2. Those who have undergone any nasopharyngeal surgery within 1 year, have a previous acute exacerbation of chronic rhinitis or have anatomical abnormalities in the nose that affect drug absorption;
3. lactating or pregnant women;
4. Those who are participating in other COVID-19-related clinical trials, and who are participating or plan to participate in other clinical trials during the study;
5. The investigators believe that the presence of any disease or condition in the close contact may put

the close contact at unacceptable risk; Close contacts are unable to meet the requirements of the program; Situations that interfere with the assessment of drug response.

## Withdrawal criteria

Withdrawal determined by the investigator :

The close contacts withdraws from the the test means that the close contacts withdraws from the test is decided by the researcher if it is not suitable to continue the test for the selected close connector. Conditions in which the researchers considered that the close contacts should not continue to participate in the experiment:

- 1. Progression of close contacts to asymptomatic or confirmed cases;
  2. The state of health does not allow continued participation in data collection, and cannot effectively ensure the safety of close contacts;
  3. Failure to promptly handle and report suspicious and unexpected serious adverse reactions; 4)Clinical trials give drugs quality problems;

5)Fraud in the course of drug clinical trials; 6)loss to follow-up;

7)Other violations of the good clinical trial practice of drugs or the investigator believes that the research cannot be continued.

Self-exit study of close contact:

According to the provisions of the informed consent form, the close contacts has the right to withdraw from the test, or the close contacts does not withdraw his informed consent, but no longer accepts the drug administration and test and loses the follow-up (also refers to withdrawal, or shedding). As far as possible, the reasons for their withdrawal should be known and documented.

## Rejection of shedding standards

Before statistical analysis of data, the main researcher and statistical analyst should judge whether the individual cases are excluded or not. In case of one of the following situations, it is necessary to comprehensively judge whether the close contacts should be excluded based on the degree to which the close contacts has completed the test and the reason for withdrawal, and make relevant explanations.

1.The selection of individual close contacts seriously violates the inclusion/exclusion criteria; 2.During the trial, close contacts did not comply with the test plan and had poor compliance, such as

not using the test medication or unable to collect biological samples according to the requirements of the test protocol, which affected the evaluation of the results.

## Termination criteria

If the investigator becomes aware of conditions or adverse events that suggest possible harm to the participant if the clinical trial continues, the decision to terminate the clinical trial may be made after appropriate consultation by the investigator. Circumstances under which clinical trials may be terminated include, but are not limited to:

- 1. The sponsor proposes to terminate the clinical trial;
  2. The NMPA or the health administrative department requires the termination of the clinical trial.

## Study site: Isolation site of Yuzhong District, Chongqing

- 1. **Test process**


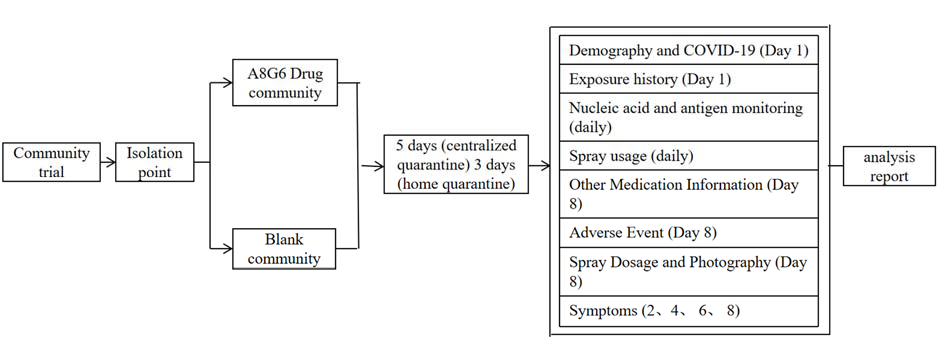
This project is a community trial, and the isolation sites that meet the requirements are selected. The isolation sites were divided into A8G6 group and blank control group. According to the inclusion and exclusion standards of the study, participants meeting the requirements of the study were included, and the informed consent was signed to collect the information of the study subjects. The specific collection process is as follows:

## A8G6 experimental group:

Test drug: A8G6 SARS-CoV-2 Neutralizing Antibody Combination Nasal Spray Specification: 5 mg/mL

At the same time, the experimental group was collected: Demography; Exposure to COVID-19;

Symptoms; Nucleic acid results; Medication compliance; Other drug information ; Spray dosage. The following is the specific information collected:

Demography: including sex, age, height, weight, occupation, education, vaccination history, Chongqing resident history, cardiovascular disease history, etc;

Exposure to COVID-19: exposure site, time, frequency and relationship with infected people; Symptoms: 16 items of symptoms related to COVID-19 patients, including cough, were scored by

0-10 numbers, where 0 was not serious and 10 was the most severe degree imaginable, and the retrospective period was within 24 hours;

Daily nucleic acid and antigen results: nucleic acid/antigen test results are positive or negative; Medication compliance: daily medication, medication interval, medication frequency and dosage; Other drug use information during centralized isolation;

Spray dosage information.

## Blank control group:

Do not use A8G6 nasal spray, but daily nucleic acid/antigen testing; And health data monitoring and recording.

## Adverse events and assessment

1. **Adverse events**

Adverse events (AE): means all adverse medical events occurring after the contact receives the test drug, which can be manifested as symptoms, signs, diseases, or abnormal laboratory tests, but are not necessarily causally related to the test drug administration. The investigator used concise language to report all adverse events observed directly or spontaneously reported by close contacts. In addition, close contacts should be asked regularly about adverse events after the trial begins.

During the trial, the adverse event record form should be filled in truthfully, including the occurrence time, severity, end time, measures taken and outcome of the adverse events, and the correlation with the study drug should be determined. Adverse events shall be recorded in the adverse events in the designated case report form.

The severity of the adverse events was judged by the NCI CTCAE 5.0 criteria. If unlisted adverse events occur, the severity of AE should also be described according to the guidelines proposed in NCI CTCAE 5.0. The guidelines are shown in Table 1:

## Table 1. Criteria for determining adverse events

| **Adverse Event classification** | **Severity description** |
| --- | --- |
| I level | Mild,no clinical or mild clinical symptoms; only clinical or laboratory abnormalities; no treatment required. |
| " level | Moderate,requiring minimal,local or non-intrusive treatment; age-appropriate limited activities of daily living (Activities of Daily Living,ADL),daily use refers to cooking,  shopping, phone calls, financial management, etc. |
| Ⅲ level | Serious or medically significant but not immediately life threatening; cause hospitalization or prolonged hospitalization; disability; restricted daily  self-care(Self care ADL).Self-care in daily life refers to:bathing, dressing,  stripping, eating,washing taking medicine,etc.,non-bedridden. |
| IV level | Life-threatening and requires emergency treatment |
| V level | Death due to an adverse event. |

According to the five criteria for the analysis of adverse reaction events in the “Adverse Drug Reaction Reporting and Monitoring Manual”(2012 edition),correlation evaluation was divided into six levels:definitely relevant, very likely relevant, possibly relevant, possibly irrelevant, to be evaluated, and not to be evaluated. Among them, adverse drug reaction definitely, likely and probably should be judged.The total number of ADR cases was taken as the numerator, and all the selected cases available for ADR evaluation were taken as the denominator to calculate the incidence of ADR. The evaluation method can be referred to Table 9.2:

## Table 2 Association evaluation of adverse events and trial drugs

| **Judgment index** | **Judgment results** | | | | | |
| --- | --- | --- | --- | --- | --- | --- |
|  | **Definitely relevant** | **Very likely relevant** | **Possibly relevant** | **Possibly irrelevant** | **To be evaluated** | **Not to be evaluated** |
| 1. Is there a reasonable |  |  |  |  |  |  |
| time relationship |  |  |  |  |  |  |
| between drug  administration and the | + | + | + | 一 |  |  |
| occurrence of adverse |  |  |  |  |  |  |
| reaction events? |  |  |  |  |  |  |
| 2 . Does the reaction |  |  |  |  |  |  |
| conform to the known  type of adverse | + | + | ± | 一 | Additional | Required |
| reactions of the drug? |  |  |  |  | material | information |
| 3.Does the reaction |  |  |  |  | required for | for |
| decrease or  disappear after the | + | + | ±? | ±? | evaluation | evaluation  is not |
| withdrawal or dosage? |  |  |  |  |  |  |
|  |  |  |  |  |  | available |
|  |  |  |  |  |  |  |
| 4. Did the same |  |  |  |  |  |  |
| reaction occur again  after the reuse of | + | ? | ? | ? |  |  |
| suspected drugs? |  |  |  |  |  |  |
| 5. Are response events  explained by concomitant medication, patient progression, and effects of other  treatments? | 一 | 一 | ±? | ±? |  |  |

Note:+ for affirmation;-for negation;± for difficult affirmation or negation;?It means the situation is unknown.

## Abnormal clinical laboratory tests and other abnormalities assessed as adverse events or serious adverse events

No clinically significant laboratory test abnormalities were not recorded as an AE or SAE. Clinically significant laboratory test abnormalities (signed as "abnormal with clinical significance", such as clinical blood routine, urine routine, blood biochemistry, coagulation function, etc.) and other abnormal assessments (e.g., electrocardiogram, vital signs, etc.) must be recorded as an AE or SAE if meeting the AE or SAE definition. If a laboratory test abnormality is part of the syndrome, the syndrome or diagnostic result (e.g. anemia) rather than the laboratory test result (i.e., hemoglobin decrease) is recorded by the investigator.

All clinical events and clinically significant laboratory test abnormalities will be treated as per the Common Adverse Event Evaluation Criteria NCI CTCAE 5.0. Both clinical events and clinically significant laboratory test abnormalities will be graded according to NCI CTCAE 5.0. Whether considered as treatment-related, all contacts with AE must be regularly monitored regularly (if feasible) until symptoms resolve, any abnormal laboratory values return to normal or return to baseline levels or are considered irreversible, or until the observed changes are properly explained. For grade 3 or 4, they should be confirmed by repeat testing if feasible.

## Serious adverse events

Serious adverse events (SAE) refer to adverse medical events such as death, life-threatening, permanent or serious disability or functional loss, hospitalization or extended hospitalization, and adverse medical events such as congenital abnormality or birth defects.

Serious and Unexpected Suspected Adverse Reaction (SUSAR): A serious and unexpected suspected adverse reaction where the nature and severity of the clinical manifestation exceeds the information available in the investigator's manual of the test drug, the instruction manual of the marketed drug or the summary of product characteristics.

## Treatment of close contact when SAE / SUSAR occurs

- 1. When considering the SAE, the principal investigator or other responsible doctors should be notified to the scene. If the condition is serious, the project leader should be notified while rescuing. If necessary, the test administration should be stopped immediately;
  2. If SAE / SAR, the close contact shall withdraw from the trial and take corresponding treatment or rescue measures according to the clinical performance; in case of massive bleeding caused by drugs, the researchers shall decide to give rescue measures to maintain the stability of the patient's vital signs as far as possible, and conduct ECG monitoring if necessary, consultation and assistance if necessary;
  3. When the close contact outside the hospital is judged to be SAE and cannot come, it is suggested that the close contact should return to the hospital or go to the local hospital in time, and immediately notify the project leader to obtain further treatment opinions; if in the local hospital, contact the receiving doctor to understand the specific situation and give treatment suggestions.

## Report of the SAE

After SAE occurs, the investigator reports according to the procedures prescribed by the clinical research institution and law.

## Data collection and follow-up

1. **Form of data collection: mainly filled in by the patient, supplemented by telephone callbacks**
   1. Can use Wechat small program: mainly to Wechat / tablet for electronic collection
   2. Those who fail to fill in the follow-up information in time: follow-up by telephone
   3. The patient is reminded by the isolation point, and the patient can scan the QR code and remind them to answer the questions
   4. Our personnel do background support, and assist the medical isolation point to provide wechat operation guidance and technical support
   5. Send short video instructions to answer questions and medication, you can check at any time.

## Frequency of data collection

During the course of the study, patients were enrolled on the day of inclusion, and the enrollment time was taken as the origin of centralized follow-up time. After the patient was released from quarantine, the basic experimental data was collected for 8 days. The observation time of related symptoms of positive patients generally lasts until the time of nucleic acid conversion, about 14 days. The specific collection frequency is as follows:

In the course of the study, patients were enrolled on the day of inclusion, and the enrollment time of patients was taken as the origin of the centralized follow-up time, and the close contact time was advanced by 1 day on this basis. After the patient is released from isolation, the basic experimental data collection is completed. The specific acquisition frequency is as follows:

| **Form content** | **Screening enrollment period** | **Isolation period** |
| --- | --- | --- |
| **1）Demography** | **√** | **/** |
| **2）History of exposure** | **√** | **/** |
| **3）Nucleic acid/antigen test results** | **√** | **Every day** |
| **4）Spray use (medication**  **only)** | **/** | **Every day** |
| **5）Other medication use**  **information** | **/** | **Day 8** |

| **6)Adverse events (medication only)** | **/** | **On Days 3, 5, and 8th** |
| --- | --- | --- |
| **7)AG86 dosage information (medication only)** | **/** | **Day 8** |
| **8)COVID-19 symptoms and body temperature questionnaire** | **√** | **On Days 2,4,6,**  **and 8** |
|  |  | **Days 2,4,6,8,10.12** |
| **9)Related symptoms of patients with positive reversion** | **√** | **and 14 after**  **positive conversion** |

## Evaluation indicators

1. **Effectiveness evaluation indicators:**

Positive conversion rate: The ratio of the number of people in close contact who turned positive (asymptomatic, confirmed cases) to the total number of people in both groups.

Median time of nucleic acid positive conversion: The median time of nucleic acid outcome of nucleic acid-positive positive patients in the two groups.

## Safety evaluation indicators:

Incidence of adverse events: Calculate the ratio of the number of patients with adverse events to the total number of patients in the three groups.

Median time of adverse events: Compare the median time to occur of adverse events in each group.

## Feasibility evaluation indicators:

Medication adherence: Compare the completeness of answers under each answer frequency in each group.

Administration time: Assess the average administration time of each does of medication in each

group

## Statistical methods

1. **Sample size calculation**

The ratio of the number of people in the A8G6 group to the control group was 1:1.Assuming a positive rate of 0.1 for the control group and 0.08 for the intervention group, the difference in conversion between the two groups is greater than 2%. It is estimated that the total sample size is about 4300, the positive conversion difference between the two groups is >2 %, the rate of missed follow-up is 20 %, with a final total enrollment of 5160.

## Baseline analysis

Measurement data: For the measurement data conforming to normal distribution, the results were expressed as mean ± standard deviation, and the A8G6 group was compared with the control group respectively. The t-test/analysis of variance was used for difference test; for measurement data that did not

conform to normal distribution, the results were expressed as median (interquartile spacing), and non-parametric test (such as rank sum test) was used for difference test.

- 1. Count data: The results of count data were expressed as the composition ratio [n(%)], and the comparison between two sample rates was performed by chi-square test or Fisher exact test.

## Effectiveness endpoints analysis:

Nucleic acid outcome rate: The chi-square test was used to compare the nucleic acid outcome rate of the closely connected population at the end of isolation or the nucleic acid outcome rate of the asymptomatic population in the two groups, respectively.

Median time to nucleic acid regression: For the time to nucleic acid outcome, the Kaplan-Meier method will be used to plot survival curves, and the Brookmeyer Crowley method will be used to calculate the 95% confidence interval of the median time. The log-rank method will also be used to compare the differences between groups and Cox proportional model will be used to estimate the HR of each endpoint and to explore the influence of other factors except the treatment group on the differences of each endpoint.

## Safety endpoints analysis.

Incidence rate of adverse events: a chi-square test was used to compare the incidence of adverse reactions in the population at the end of isolation in both groups.

Median time of adverse events: The Kaplan-Meier method will be used to plot survival curves, and the Brookmeyer Crowley method will be used to calculate the 95% confidence interval of the median time. The log-rank method will be used to compare the differences between groups.

## Comparison of trajectories of symptom relief and quality of life changes among the groups.

Symptom burden description: mean, standard deviation, median, maximum, minimum, P25, P75 were used to describe the symptom profile of the surveyed population, and frequency and percentage (N, %) were used to describe the proportion of patients with severe symptoms and quality of life.

Symptom recovery to normal level: K-M (Kaplain-Meier) method or life table method was used for calculation, log-rank test was used for comparing the difference between groups and plotting recovery curve.

Inter-group comparison of the trajectory of symptom change between two groups: mixed-effects model and generalized estimating equation were used to compare the incidence of symptoms and moderate to severe symptoms between groups, respectively.

## Medication adherence, medication time cost:

Medication adherence and time cost of the intervention groups were analyzed using mean ± standard deviation or response completeness and missing rate descriptively.

## Trial drug management

## Trial drug information

Trial drug: A8G6 SARS-CoV-2 Neutralizing Antibody Combination Nasal Spray Specification: 5 mg/mL

Provided by Chongqing Mingdao Hoyue Biotechnology Co., Ltd. The label style used for the test is as follows：

|  | For clinical trial use only Keep out of reach of children  Nasal Spray C  【Batch No.】C-20221103  【Package specification】6 mL/bottle  【Storage conditions】2-8℃ storage  【Dosage】Nasal spray, 1 dose for each nostril  【Expiration date】Until February 1, 2024  【Applicant】Chongqing Mingdao Hoyue Biotechnology Co., Ltd. |  |
| --- | --- | --- |

## Receipt, distribution and storage of experimental drugs

The experimental drugs shall be randomly drawn from the production line in accordance with GMP standards, and shall pass the test. Chongqing Mingdao Haoyue Biotechnology Co., Ltd. shall provide clinical research institutions with adequate quantities of qualified test drugs, marked with their batch numbers, and provide drug inspection reports. The quantity shall meet the requirements of the test and drug retention quantity.

The research institution shall sign the drug receipt form when receiving the drug for test, check and accept it on the spot and sign it. Experimental drugs shall be stored in accordance with the storage conditions of experimental drugs, drug management shall follow the requirements of GCP[6], and the research institution shall appoint special personnel and counters for storage. Investigational drugs are intended for clinical trials only and are not intended for use in any other situation. At the end of the study, the inspector checks all unused drugs.

## Method of administration for close contacts

Intranasal administration, one spray to the left and right nostrils, as 1 dose. Three doses were administered every day, with a 6-8 hour interval for 5 days. (See instructions and videos for specific operations)

## Medication compliance

In the stage of recruitment and screening of close contacts, the researcher shall introduce in detail the purpose of this experiment, basic information of experimental drug administration, research protocol, test process, drug administration scheme (such as dosage, method of administration, etc.), clinical observation, frequency and process of biological sample collection, and potential risks of participating in the experiment. Make the contacts fully informed, voluntary participation, improve the drug administration compliance. Before administration, the investigator should carefully check the cipher number, dosing random code table, dosing dosage and order; After administration, make a careful inventory of the remaining quantities, empty packages, and instruments of administration.

The study drug will be administered under direct medical supervision, and the investigator will accurately record the administration in the original record (study medical record or close contact drug release sheet).

## Preservation and confidentiality of test documents

## Preservation of test files

In order to ensure the evaluation and supervision of clinical studies by the State Medical Products Administration and the sponsor, the investigator shall agree to keep all study data, including the original record form of the close contact test, informed consent form, case report form, detailed records of drug distribution, etc. The paper and electronic documents generated by the laboratory and sample testing laboratory should be properly stored to ensure timely traceability. Investigators should keep trial data for at least 5 years after the drug is approved for marketing. The sponsor shall keep clinical trial data for at least 5 years after the drug is approved for marketing. The ownership of all data of this clinical study belongs to the sponsor, and the documents of the laboratory shall not be damaged without prior written agreement between the investigator and the sponsor. If the investigator chooses to provide the test documents to another party or transfer them elsewhere, the sponsor must be notified. Unless required by the State Medical Products Administration, the researcher shall not provide the information in any form to a third party without the written consent of the sponsor.

## Confidentiality of test documents and data publication

The researcher must keep the contact confidential. The researcher shall establish a separate password file, including the password number, name and address, which shall be kept in strict confidence by the research institution. Case reports and other documents to be delivered to the sponsor shall be identified by code only, not by name.

Data provided to the investigator by the sponsor (including this clinical trial protocol) is non-public information and must be kept confidential. Without the written permission of the sponsoring unit and the person in charge of the clinical trial institution, no one shall disclose the data to others in any way. Units and individuals violating these Provisions will be investigated for their responsibilities and punished according to relevant provisions.

## Quality control and guarantee of research

In this study, specialized personnel will be designated for data management and data management plan will be formulated. Regular data monitoring will be conducted during the acquisition of PRO and clinical indicators data. RWD data platform will be adopted for data management to ensure data traceability.

## Data Collection

All data collectors are required to receive training before starting data collection, including: use of spray medication, patient data collection methods, PRO symptom investigation methods, data collection quality control, etc.

## Data Verification

Compare the data in the database with the flow control report in a combination of manual verification and computer assistance, check the results that are different from the flow control report, and then check item by item with the original flow control report table to make corrections, so as to ensure that the data in the database is consistent with the results in the flow control report.

## Data Modification

RWD system and small program with trace tracking function, every modification of data will leave the modification and modification time information, convenient scientific research supervision department to supervise the project implementation process.

## Problem handling in the research

The technical problems found in the regular research progress meeting and verification shall be discussed by the expert committee of the project consultant, put forward solutions and coordinate the implementation. If quality problems are found, relevant studies will be suspended, a comprehensive audit will be conducted, corrective suggestions will be put forward, and relevant personnel will be educated and dealt with.

## Data security monitoring

The data collected in this study contains a large number of patients' personal and health information, which has certain data security risks. In order to ensure patient information security, project information management measures will be developed. The main principles include:

- 1. In accordance with the National Medical Products Administration 2020 "Real World Data for Clinical Evaluation of medical devices Technical Guidelines (Trial)", formulate data standards and safety rules, adopt PI responsibility system, scientific research departments play the implementation of security and supervision responsibilities, and specify the whole process of data collection, cleaning, integration, analysis and reporting. Participants sign confidentiality agreements, receive supervision, and have zero tolerance for any behavior and personnel violating data security regulations.
  2. Chongqing Medical University shall pass the ethical review of data collection and analysis, encrypt relevant data, and blind patients' personal information to data analysts, so as to avoid the ethical risk of patient information disclosure.
  3. Each database in the RWD system is managed by researchers themselves or designated personnel, and the user name and password are used to determine the permission of researchers or managers, which ensures data security and the convenience of data extraction for researchers.
  4. A dedicated data collection platform is used, which is located in the firewall of the subject undertaking unit. Data collection and transmission are connected with the data platform through telecom cloud.
  5. Invite domestic and foreign experts in disease control, symptom research, data management, machine learning and statistical analysis to set up a technical advisory committee, hold regular project technical advisory meetings, give professional guidance in the whole process from design to report, and constantly improve the design and methods according to the problems encountered in the implementation of the project.

## Research progress

| Time arrangement | Research task | Main objective |
| --- | --- | --- |
| 2022.11.15-2022.11.21 | Research data preparation, drug preparation, preliminary research plan and implementation  rules | Determine the project plan |
| 2022.11.22-2022.11.26 | Design data collection platform and conduct investigator training | Build and debug the platform |

| 2022.11.27-2023.08.31 | The experiment was initiated, patients were enrolled and followed up, and the follow-up data were collected by electronic questionnaire | Execution plan |
| --- | --- | --- |
| 2023.09.01-2023.12.31 | Data analysis and report writing | Complete the main results report and article  writing |

## Participants

| Name | Major | Technical title | Assignment |
| --- | --- | --- | --- |
|  |  |  |  |
|  |  |  |  |
|  |  |  |  |
|  |  |  |  |
|  |  |  |  |
|  |  |  |  |
|  |  |  |  |
|  |  |  |  |
|  |  |  |  |
|  |  |  |  |
